# Generation of bat-derived influenza viruses and their reassortants

**DOI:** 10.1038/s41598-018-37830-x

**Published:** 2019-02-04

**Authors:** Masahiro Sato, Junki Maruyama, Tatsunari Kondoh, Naganori Nao, Hiroko Miyamoto, Yoshihiro Takadate, Wakako Furuyama, Masahiro Kajihara, Hirohito Ogawa, Rashid Manzoor, Reiko Yoshida, Manabu Igarashi, Ayato Takada

**Affiliations:** 1 Division of Global Epidemiology, Research Center for Zoonosis Control, Hokkaido University, Sapporo, Japan; 2 Department of Virology, Okayama University Graduate School of Medicine, Dentistry and Pharmaceutical Sciences, Okayama, Japan; 3 Global Station for Zoonosis Control, Global Institution for Collaborative Research and Education, Hokkaido University, Sapporo, Japan; 4 School of Veterinary Medicine, the University of Zambia, Lusaka, Zambia; 5 Present Address: Department of Pathology, The University of Texas Medical Branch, Galveston, Texas USA; 6 Present Address: Department of Virology 3, National Institute of Infectious Diseases, Musashimurayama, Japan; 7 Present Address: Laboratory of Virology, Division of Intramural Research, National Institute of Allergy and Infectious Diseases, National Institutes of Health, Rocky Mountain Laboratories, Hamilton, Montana USA

## Abstract

Two novel influenza A virus-like genomes were detected in fruit bats in Central and South America. However, the biological properties of these bat-derived influenza viruses (BatIVs) are still largely unknown since infectious viral particles have never been isolated from the infected host species. In this study, a reverse genetics approach was used to generate infectious BatIV particles entirely from plasmids encoding full-length sequences in eight gene segments. We inoculated BatIV particles into various cell cultures including bat-derived cell lines and found that BatIVs infected particular bat-derived cells efficiently but not the other cell lines tested. Reassortant viruses between the two BatIVs were also successfully generated and their replication in the susceptible bat cell lines was confirmed. These findings suggest a limited host range and reassortment potential of BatIVs in nature, providing fundamental information for understanding of the ecology of BatIVs.

## Introduction

Influenza A viruses (IAVs), which belong to the family *Orthomyxoviridae*, cause highly contagious diseases in a wide variety of avian and mammalian species, including humans, pigs, horses, dogs, and poultry, and are recognized as one of the most important zoonotic pathogens. IAVs have 8 segmented negative-sense RNA genomes and are divided into subtypes based on combination of two viral envelope glycoproteins, hemagglutinin (HA) and neuraminidase (NA). IAVs with H1-16 HA and N1-9 NA subtypes have been identified in wild aquatic birds, especially migratory ducks, the natural reservoir of IAVs^1–4^. Importantly, due to their propensity for genetic reassortment, a variety of IAV subtypes are distributed in many host species.

Recently, influenza virus-like RNA genomes were detected in fruit bats (*Sturnira lilium* and *Artibeus planirostris*) in Central and South America^5,6^. Since the amino acid sequences of their HA and NA are distinct from those of all previously known IAV subtypes, these bat-derived influenza viruses (BatIVs) were provisionally designated H17N10 and H18N11. However, previous studies have reported that H17 HA does not bind to sialic acids linked to galactose in sugar chains^7^, which are known as canonical receptors of IAVs, and that N10 NA lacks neuraminidase activity^8^ which is also a common property of all known influenza viruses. Thus, BatIV glycoproteins have also been called HA-like (HL) and NA-like (NL) (i.e., HL17NL10 and HL18NL11)^9^. However, information on the biological properties of these BatIVs is limited since infectious virus particles have never been isolated from infected animals.

Bats belonging to the order Chiroptera, which is known as the second largest order of mammalians, are distributed into more than 1,000 species globally^10^. It has been shown that bats play crucial roles as natural reservoirs of some zoonotic pathogens such as Marburg virus, Hendra virus, Nipah virus, Lyssa virus, SARS, and MERS coronaviruses^11–14^. Thus, it is important to investigate the ecology of bat-derived pathogens and to clarify their potential risks as zoonotic pathogens.

Our previous study revealed that vesicular stomatitis viruses (VSVs) pseudotyped with BatIV glycoproteins efficiently infected cultured cells derived from particular bat species (e.g., *Miniopterus fuliginosus*) but not those commonly used for IAV propagation and other bat cells tested, providing key information on cell lines that are potentially susceptible to BatIVs^15^. Using these bat cell lines and the well-known plasmid-based reverse genetics approach^16^, we demonstrate the generation of infectious BatIVs and their reassortants in this study.

## Results

### Generation of BatIV from plasmids

**Figure 1:**
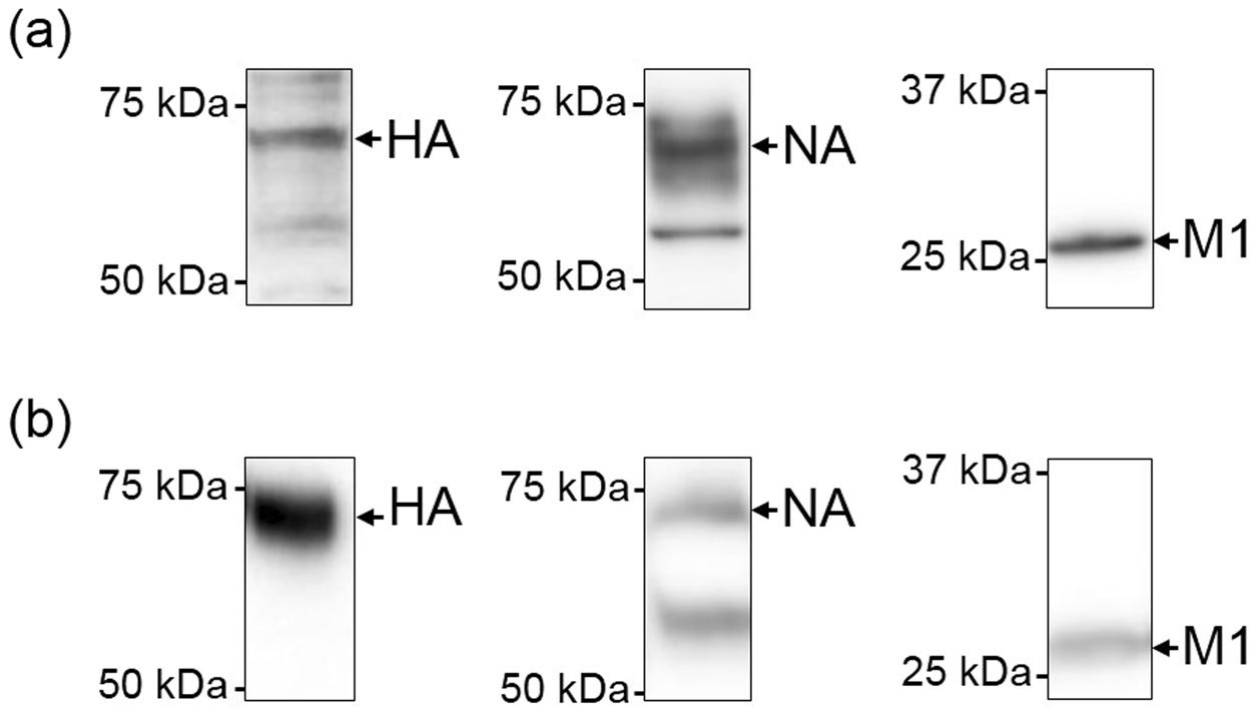
Detection of viral proteins of H17N10 BatIV in the cell lysate and supernatant. HEK293T cells transfected with plasmids were cultured for 48 hours at 37 °C. After incubation, cell lysates (**a**) and supernatants (**b**) were harvested for western blotting to detect H17 HA, N10 NA, and M1 proteins as described in Materials and Methods.

We first attempted to generate H17N10 BatIV by transfecting human embryonic kidney (HEK) 293T cells with 8 pol-I plasmids providing viral RNA templates and 4 pCAGGS plasmids expressing the viral nucleoprotein (NP) and polymerases required for viral RNA transcription/replication. To confirm the expression of HA, NA, and viral matrix protein (M1) in the transfected cells, cell lysates and supernatants were analyzed by western blotting. We detected H17 HA, N10 NA, and M1 as approximately 70 kDa, 70 kDa, and 27 kDa bands, respectively, in the cell lysates (Fig. 1a), suggesting that plasmid-driven viral RNA transcription/replication successfully occurred in the transfected cells. We further confirmed that these viral proteins were detectable in the supernatants of the transfected cells (Fig. 1b). Pol-I and pCAGGS plasmids providing viral RNA and proteins of H18N11 BatIV were also constructed and similarly used for generation of the virus.

### Morphology of BatIV particles

**Figure 2:**
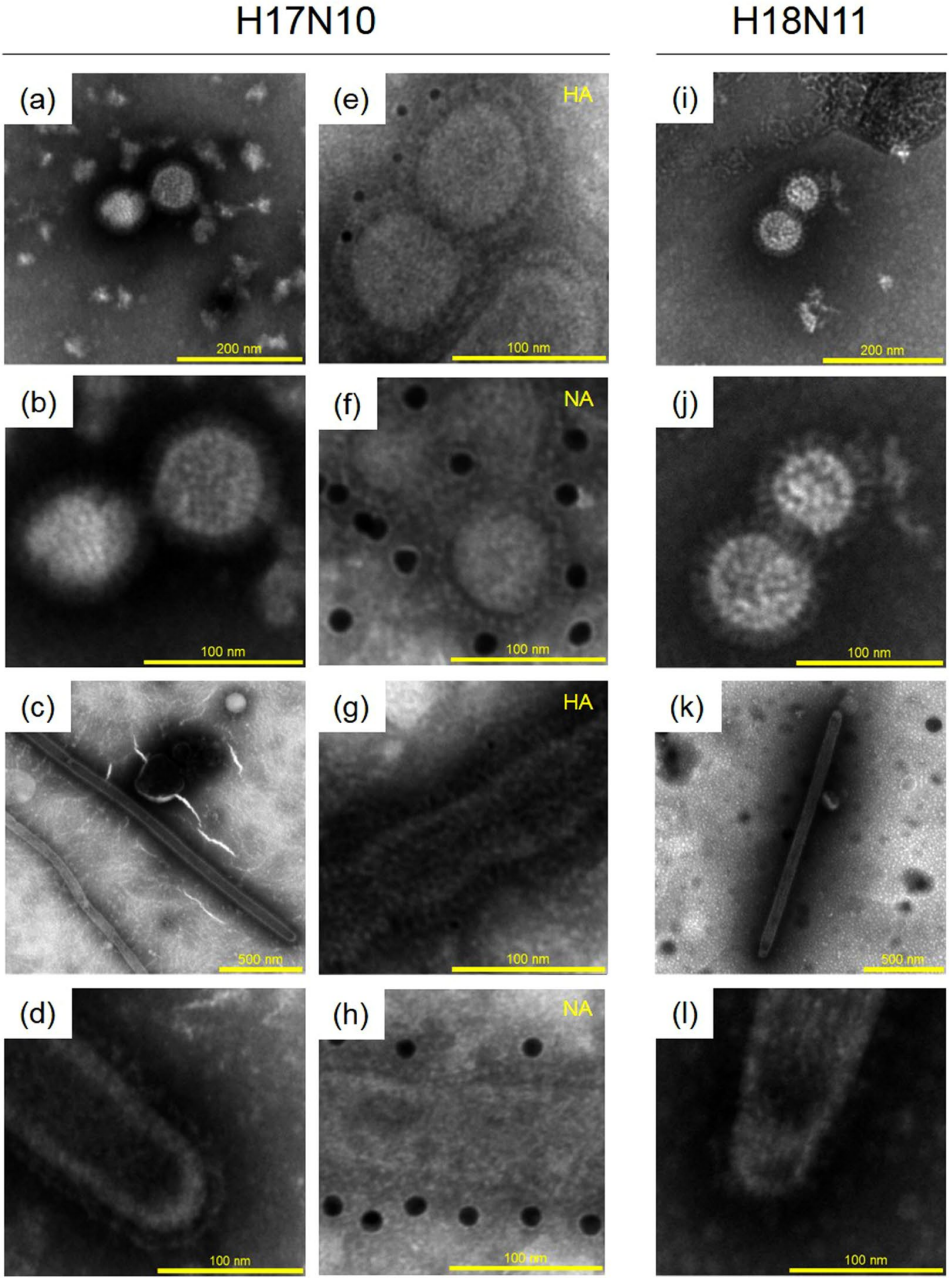
Morphology of BatIV particles. H17N10 (**a**–**h**) and H18N11 (**i**–**l**) virions in the supernatant were fixed and negatively stained as described in Materials and Methods. For immune transmission electron microscopy, an anti-HA2 monoclonal antibody (**e**,**g**) and anti-N10 NA rabbit serum (**f**,**h**) were used.

To observe viral particles in the supernatants of the transfected cells, we used transmission electron microscopy (TEM) and confirmed the presence of virions with numerous spikes on their surfaces. Interestingly, there were two morphologically different virus particles with spherical and long filamentous structures (Fig. 2). The virion diameter of the spherical particles was approximately 100 nm (the average and standard deviation of 30 randomly selected particles was 105 ± 23 nm, which was similar to that of typical IAV, A/Puerto Rico/8/1934 (H1N1) (96 ± 22 nm). On the other hand, the virion length of the long filamentous particles was over 1,000 nm. Densely arrayed spike structures were observed on the surfaces of both viral particles. Immuno-TEM revealed that these spikes were BatIV glycoproteins, H17 HA and N10 NA proteins (Fig. 2e–h and Supplementary Fig. 1). No significant difference was found in the overall morphology between H17N10 and H18N11 BatIV particles.

### Infection of YubFKT2 cells with BatIV

**Figure 3:**
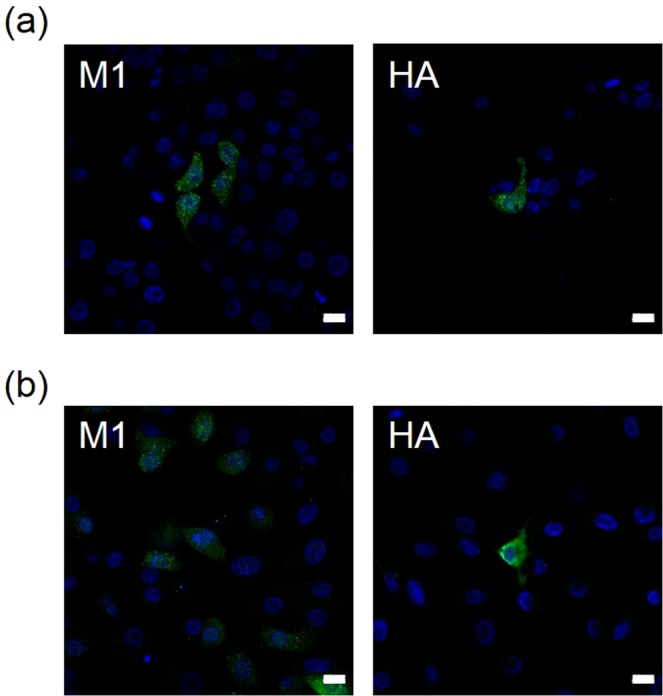
BatIV protein synthesis in bat cells detected by IFA. H17N10 BatIV particles in the supernatant of transfected HEK293T cells were concentrated, treated with trypsin and inoculated into YubFKT2 (**a**) and SuBK12-08 (**b**) cells. Forty-eight hours later, cells were fixed and stained for detecting M1 and HA proteins. Scale bars represent 10 µm.

To confirm the potential of generated BatIV particles to infect cells, we first used the cell line YubFKT2, which is derived from the bat (*M. fuliginosus*). BatIV particles were treated with trypsin to cleave the HA protein, which is generally required for activation of IAV HAs, and inoculated into YubFKT2 cells. Forty-eight hours later, the cells were fixed and stained for detecting M1 and HA proteins in immunofluorescent assays (IFA) (Fig. 3). We found that infected cells were clearly stained with the antibodies specific to these viral proteins, suggesting that viral RNA transcription/replication followed by viral protein synthesis occurred in these cells.

### Cell lines susceptible to BatIV

**Figure 4:**
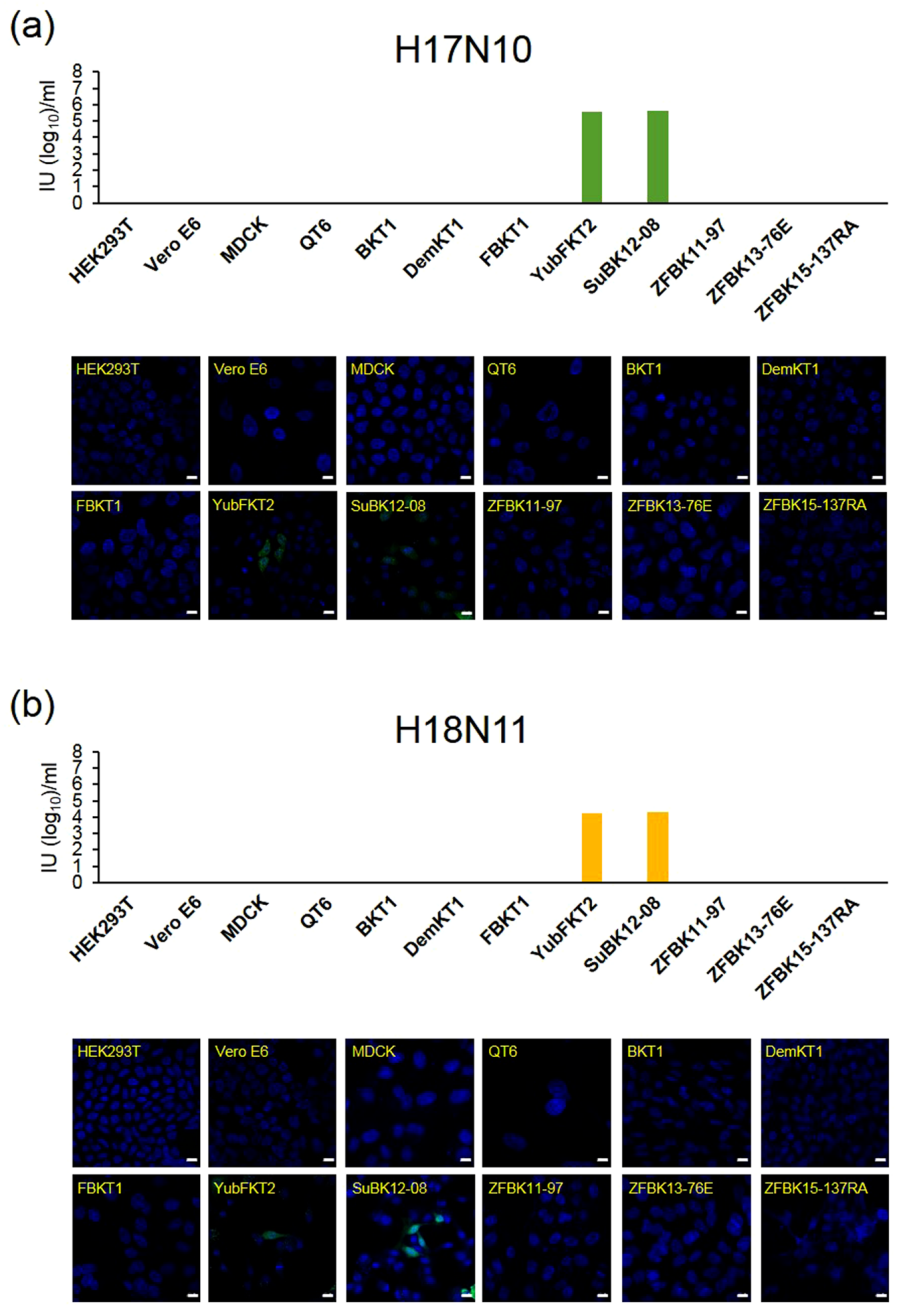
Infectivities of BatIVs in various cell lines. H17N10 (**a**) and H18N11 (**b**) BatIVs were inoculated into various cell lines. Infectious units (IUs) of the viruses in different cell lines were determined by counting the number of IFA-positive cells stained with the anti-M1 monoclonal antibody. Scale bars represent 10 µm. Experiments were triplicated and the representative data are shown.

In a previous study, we screened various cell lines, including bat-derived cells, for their susceptibility to VSV pseudotyped with BatIV glycoproteins and found that the virus infected particular bat cell lines^15^. To confirm this tropism, BatIV particles produced from the transfected HEK293T cells were inoculated into various cultured cells (i.e., 1 avian and 11 mammalian cell lines including 8 that were derived from bats) (Table 1) and the cells were stained for IFA. Among the bat-derived cell lines, the *Miniopterus fuliginosus* and *Miniopterus schreibersii* bat-derived lines (YubFKT2 and SuBK12-08 cells, respectively) showed susceptibility to BatIV. However, no infected cells were observed in the other mammalian and avian cell lines, including Vero E6, MDCK, and QT6. To quantify the infectivity of BatIV in each cell line, we calculated infectious units by counting the number of fluorescent cells and found that both H17N10 and H18N11 BatIVs showed similar preference profiles of infectivity (Fig. 4a,b). Interestingly, YubFKT2 and SuBK12-08 cells showed susceptibility equivalent to or higher than MDCK II cells which have been reported to be susceptible to BatIVs (Supplementary Fig. 2)^17^.

**Table 1:** Origins of cell lines used in this study.

Cell lines	Origins	Species
HEK293T	Human	*Homo sapiens*
Vero E6	African green monkey	*Chlorocebus* sp.
MDCK	Dog	*Canis lupus familiaris*
MDCK II	Dog	*Canis lupus familiaris*
QT6	Japanese quail	*Coturnix coturnix japonica*
BKT1	Greater horseshoe bat	*Rhinolophus ferrumequinum*
DemKT1	Leschenault’s rousettus	*Rousettus leschenaultii*
FBKT1	Yaeyama flying fox	*Pteropus dasymallus yayeyamae*
YubFKT2	Eastern bent-winged bat	*Miniopterus fuliginosus*
SuBK12-08	Schreiber’s bat	*Miniopterus schreibersii*
ZFBK11-97^a^	Peters’s epauletted fruit bat	*Epomophorus crypturus*
ZFBK13-76E	Straw-colored fruit bat	*Eidolon helvum*
ZFBK15-137RA	Egyptian fruit bat	*Rousettus aegyptiacus*

^a^Temporarily determined by habitat and nucleotide sequence identity of cytochrome *b* genes (97% in BLAST search). East African epauletted fruit bat (*Epomophorus minimus*), Ansell’s epauletted fruit bat (*Epomophorus anselli*), Peter’s dwarf epauletted fruit bat (*Micropteropus pusillus*) and Gambian epauletted fruit bat (*Epomophorus gambianus*) are also genetically similar (97%).

### Generation of BatIV reassortants from plasmids

**Figure 5:**
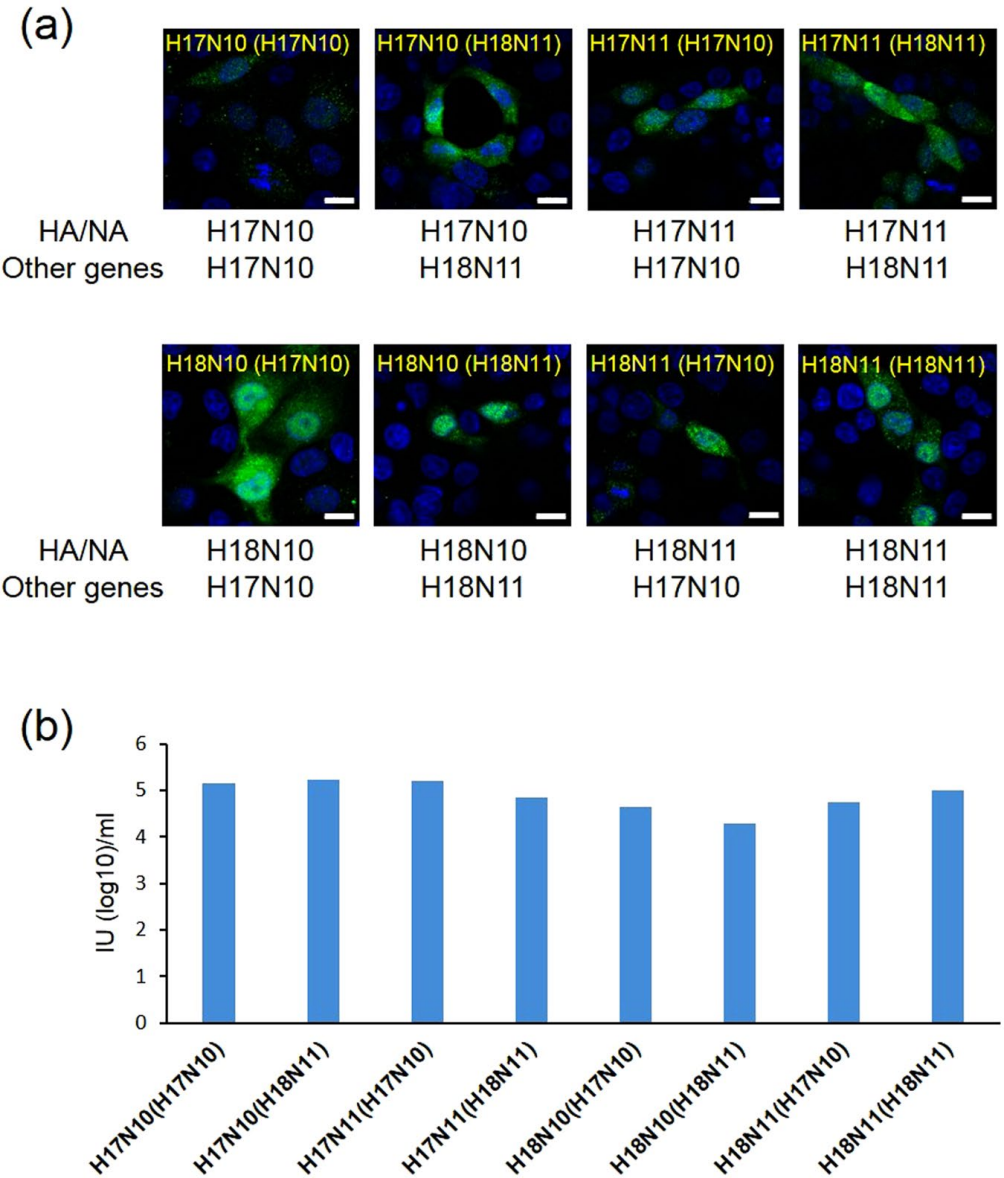
Infectivities of BatIV reassortants. (**a**) Reassortant viruses consisting of H17 or H18 HA genes, N10 or N11 NA genes, and internal genes of H17N10 or H18N11 were produced with many combinations. Reassortant viruses were inoculated into YubFKT2 cells. Forty-eight hours later, the cells were fixed and stained for the detection of the M1 protein. Scale bars represent 10 µm. (**b**) Infectious units (IUs) of the viruses were determined by counting the number of IFA-positive cells stained with the anti-M1 monoclonal antibody. Experiments were triplicated and the representative data are shown.

We further attempted to generate reassortants between the two BatIVs. HEK293T cells were transfected with plasmids for reassortant viruses whose NA segments were swapped with each other (i.e., H17N11 and H18N10) and those having H17 HA and N10 NA segments with internal protein gene segments of H18N11 BatIV and vice versa. We confirmed that all these reassortant viruses were successfully generated and efficiently infected YubFKT2 cells (Fig. 5).

## Discussion

Infectious BatIVs have never been isolated from bats^5,6^. Thus, reverse genetics approaches have been used to create recombinant BatIVs. However, some previous studies could only investigate reassortant viruses containing BatIV internal protein gene segments and HA/NA gene segments of well-characterized IAVs and failed to generate wild-type BatIVs, most likely due to the strict host cell specificity (i.e., receptor usage) for propagation of BatIVs^18,19^. Recently, one research group confirmed the rescue of infectious BatIV with its whole gene segments using the cell line MDCK II which originated from highly passaged MDCK cells and has different properties from the parent cell line commonly used for IAV propagation^17^. In this study, we demonstrated the generation of infectious BatIVs and their reassortants using HEK293T cells.

Consistent with the previous study^17^, BatIV proteins were newly synthesized in plasmid-transfected HEK293T cells, indicating that the BatIV ribonucleoprotein complex worked in human kidney cells, leading to the production of progeny virus particles. Interestingly, we found two morphologically different viral particles in the supernatant of the transfected cells. It is known that virions of IAVs isolated from clinical specimens often have spherical and filamentous shapes^20^. However, it is unclear whether the infectious potential of BatIVs depends on the morphological differences of virions. Further studies are required to clarify the relation between virion morphology and viral infectivity.

We generated reassortant viruses of BatIVs (e.g., H17N11 and H18N10). This is the first report of reassortants generated between the two BatIVs that have been discovered so far, indicating that these BatIVs share their packaging signals in their RNA genomes. It is thought that each RNA segment contains packaging signals within the 3′ and 5′ non-coding and parts of coding regions in the genomic RNA^21^. Previous studies tried to generate reassortant viruses consisting of BatIV segments and those of other previously known IAVs^18,19^. Although BatIV segments have almost identical sequences at the extreme 3′ and 5′ ends, these attempts failed to generate reassortant viruses. However, BatIV-based chimeric viruses whose HA and NA segments have the BatIV non-coding region with adjacent parts of the coding region of well-characterized H1 and H7 HAs were generated in previous studies^18,19^. It was also suggested that the failure of BatIV reassortants with other known IAVs was due to the incompatibility of NP and genomic segments^22^. This evidence implied that the non-coding region and adjacent parts of the coding region might also play critical roles in genome packaging in BatIVs. Taken together, our results suggest the potential for reassortment among BatIVs in nature, though there may be little possibility that BatIVs generate reassortant viruses with avian and mammalian IAVs.

In this study, cells were inoculated with BatIVs from an apical direction. A previous study reported that BatIVs infected preferentially at the basolateral membrane of MDCK II cells^17^. In this study, since BatIV particles were inoculated into sub-confluent cells, it might be possible that infection was initiated at basolateral side, especially the edge of monolayer cells. Alternatively, there may be a difference of expression and localization of BatIV receptor molecule(s) between susceptible bat-derived cell lines and MDCK II cells. It would be of interest to investigate infection through the basolateral site of bat-derived cells. More importantly, BatIV cell tropism is likely determined by the receptor engagement of the virus since the cell lines susceptible to BatIVs were consistent with those of VSVs pseudotyped with BatIV surface glycoproteins^15^. It has been reported that HAs of BatIVs do not recognize sialic acids, which are known as the receptor molecules of other known IAVs^23^. In addition, crystal structure analysis of BatIV HAs revealed that the receptor binding pocket of BatIV HAs is smaller than those of the other IAVs examined^23,24^. Although the receptor molecules of BatIVs are still unknown, a previous study indicated that BatIV receptor(s) may be a certain glycoprotein shared by some particular bat species^15^.

Although we confirmed the infectiveness of BatIV particles by a single-step replication cycle of BatIVs in individual cells, it is unclear that these bat-derived cell lines allow multiple replication cycles of the virus in the presence of trypsin. Like previously known avian and mammalian IAVs, BatIVs also require HA cleavage activation with trypsin-like proteases for their entry into cells^15^. However, since the two BatIV-susceptible cell lines used in this study were highly sensitive to trypsin and also unable to be maintained in fetal calf serum (FCS)-free media, these cell lines could not be used for the cultivation of the virus in the conditions for multiple replication cycles *in vitro*. Our long-term goals are to biologically characterize BatIVs, identify receptor molecules of BatIVs, and elucidate the host range and ecology of BatIVs in nature. Once information on the cellular receptor molecule(s) for BatIVs is in hand, it may be possible to generate fully susceptible cell lines by introducing the molecule(s) and thus to prepare large amounts of infectious BatIV particles needed to fully characterize their biologically properties both *in vitro* and *in vivo*.

## Methods

### Cells

HEK293T and Vero E6 cells were grown in Dulbecco’s modified Eagle’s medium (DMEM) supplemented with L-glutamine, 10% FCS, 100 U/ml penicillin, and 0.1 mg/ml streptomycin. MDCK cells were grown in DMEM supplemented with L-glutamine, 10% calf serum, and penicillin-streptomycin. QT6 cells were grown in F-12K medium supplemented with 5% FCS, 5% TPB, and penicillin-streptomycin, MDCK II cells were grown in Eagle’s minimum essential medium supplemented with L-glutamine, 5% FCS. All bat-derived cell lines were grown in RPMI-1640 medium supplemented with L-glutamine, 10% FCS, and penicillin-streptomycin^25,26^.

### Construction of plasmids

All segments of H17N10 and H18N11 BatIV genes (GenBank Accession numbers CY103881-CY103889 and CY125942-CY125949) were amplified using plasmids encoding each gene segment (kindly provided from Dr. Suxiang Tong) and cloned into the Pol-I plasmid pHH21 as described previously^16^, using Gibson Assembly Master Mix (New England Bio Lab). Viral polymerase (i.e., PB2, PB1, and PA) and NP genes were cloned into the protein expression vector pCAGGS^27^.

### Generation of BatIVs and their reassortants

HEK293T cells (4.0 × 10^5^) were seeded into 6-well plates and transfected with eight Pol-I plasmids (0.1 μg for each segment) and four protein expression plasmids (1.0 μg for PB2, PB1, PA, and NP) using TransIT-LT1 (Mirus Bio LLC) according to the manufacturer’s protocol. Supernatants of the transfected cells were harvested at 48 hours post-transfection and were centrifuged through a 25% sucrose cushion (28,000 rpm, 2 hours, 4 °C). Concentrated (200X) virus particles were treated with 5.0 μg/ml trypsin for 1 hour at 37 °C and then inoculated into YubFKT2 and other cells seeded on 6-well plates.

### Western blotting

Sodium dodecyl sulfate-polyacrylamide gel electrophoresis (SDS-PAGE) and western blotting were conducted as described previously^15^. Briefly, concentrated BatIV particles were mixed with SDS-PAGE sample buffer with 5% 2-mercaptoethanol and boiled for 5 minutes. After electrophoresis on 5–20% SuperSep Ace (Wako), separated proteins were blotted on a polyvinylidene difluoride membrane (Millipore). The membrane was incubated with a mouse anti-M1 monoclonal antibody (APH 6-23-1-6)^28^, a mouse anti-HA2 monoclonal antibody produced in our laboratory (H13N6 148-6-6) or anti-N10 NA rabbit polyclonal antibody (FS0181) recognizing amino acid positions 328–343 (AQEKGEGGIQGFILDE) followed by incubation with peroxidase-conjugated goat anti-rabbit IgG (H + L) or goat anti-mouse IgG (H + L) (Jackson ImmunoResearch). The bound antibodies were visualized with Immobilon Western (Millipore).

### Transmission electron microscopy (TEM)

BatIV particles fixed with 0.25% glutaraldehyde were adsorbed onto collodion-carbon-coated copper grids and negatively stained with 2% phosphotungstic acid solution (pH 5.8). For immuno-TEM, we used an anti-HA2 mouse monoclonal antibody (H13N6 148-6-6) and anti-N10 NA rabbit polyclonal antibody (FS0181) as primary antibodies and immunogold-conjugated goat anti-mouse IgG (H + L) 5 nm Gold (BB International) and goat anti-rabbit IgG (H + L) 15 nm Gold (Abcam) antibodies. Samples were examined with an H-7650 electron microscope (Hitachi) at 80 kV.

### Immunofluorescent assay

Forty-eight hours after inoculation of BatIV, the cells were fixed with 4% formalin in phosphate-buffered saline (PBS) for 20 minutes. They were then blocked with 2% bovine serum albumin/PBS overnight at 4 °C and then permeabilized for 7.5 minutes using 0.2% Triton X-100/PBS. The cells were stained with anti-M1 (APH 6-23-1-6) or anti-HA2 (H13N6 148-6-6) monoclonal antibodies and an Alexa Fluor 488-conjugated anti-mouse secondary antibody (Invitrogen). Samples were observed with an LSM780 confocal microscope (Carl Zeiss).

## Supplementary information

Supplemental Figures
