# Supplementary material for: Generation of bat-derived influenza viruses and their reassortants
Source: Sci Rep. 2019 Feb 4;9:1158. doi: 10.1038/s41598-018-37830-x (PMC6362294; doi:10.1038/s41598-018-37830-x)
Supplement: Supplementary file 1 — Supplemental Figures [file 41598_2018_37830_MOESM1_ESM.pdf]

Generation of bat-derived influenza viruses and their reassortants

Masahiro Sato[1], Junki Maruyama[1]†, Tatsunari Kondoh[1], Naganori Nao[1]†, Hiroko Miyamoto[1], Yoshihiro Takadate[1], Wakako Furuyama[1]†, Masahiro Kajihara[1], Hirohito Ogawa[2], Rashid Manzoor[1], Reiko Yoshida[1], Manabu Igarashi[1,3] & Ayato Takada[1,3,4*]

[1]Division of Global Epidemiology, Research Center for Zoonosis Control, Hokkaido University, Sapporo, Japan

[2]Department of Virology, Okayama University Graduate School of Medicine, Dentistry and Pharmaceutical Sciences, Okayama, Japan

[3]Global Station for Zoonosis Control, Global Institution for Collaborative Research and Education, Hokkaido University, Sapporo, Japan

[4]School of Veterinary Medicine, the University of Zambia, Lusaka, Zambia

†Current address

Junki Maruyama: Department of Pathology, The University of Texas Medical Branch, Galveston, Texas, USA

Naganori Nao: Department of Virology 3, National Institute of Infectious Diseases, Musashimurayama, Japan

Wakako Furuyama: Laboratory of Virology, Division of Intramural Research, National Institute of Allergy and Infectious Diseases, National Institutes of Health, Rocky Mountain Laboratories, Hamilton, Montana, USA

[*]atakada@czc.hokudai.ac.jp

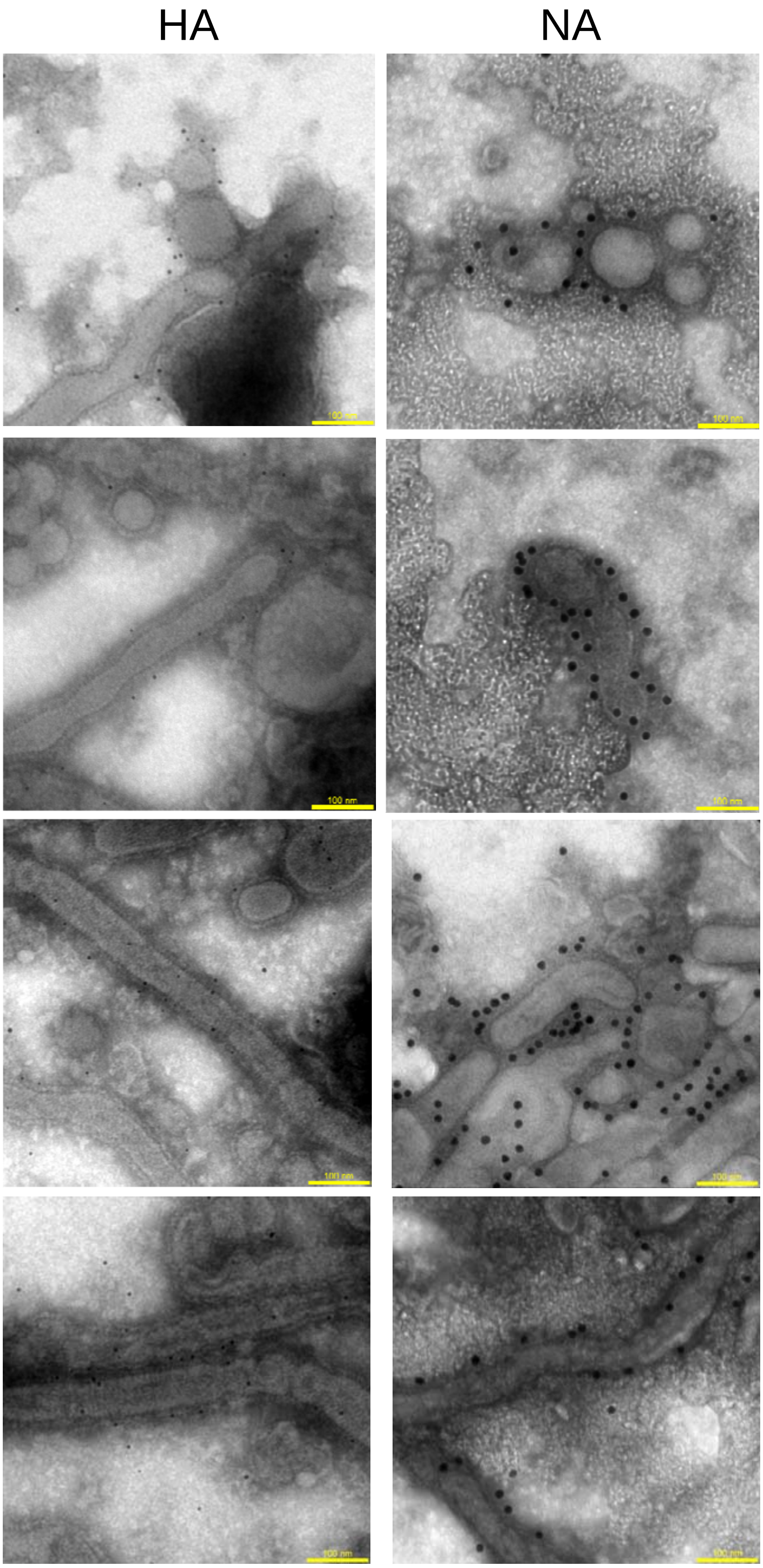

Supplementary Fig. 1. Immune transmission electron microscopy of BatIV H17N10 particles. An anti-HA2 monoclonal antibody and anti-N10 NA mouse serum were used as described in Materials and methods. Scale bars represent 100 nm.

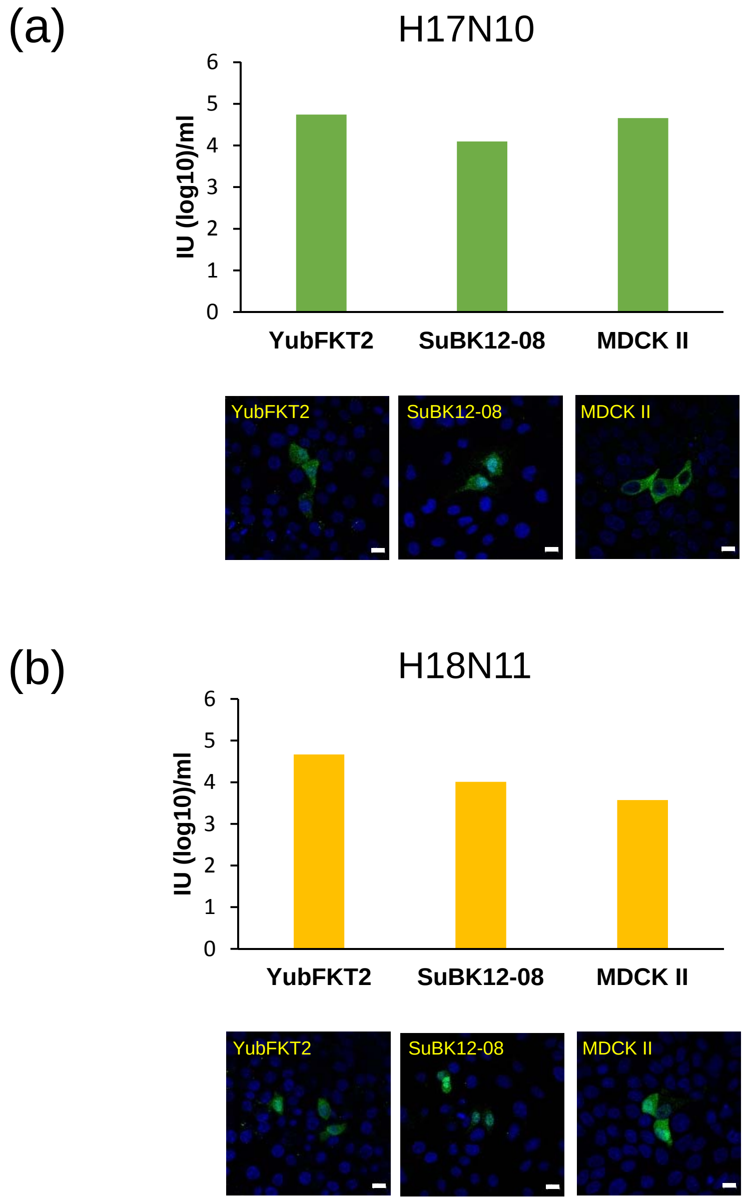

Supplementary Fig. 2. Infectivities of BatIVs in bat and MDCK II cell lines. H17N10 (a) and H18N11 (b) BatIVs were inoculated into YubFKT2, SuBK12-08, and MDCK II cell lines. Infectious units (IUs) of the viruses in different cell lines were determined by counting the number of IFA-positive cells stained with the anti-M1 monoclonal antibody. Scale bars represent 10 µm. Experiments were triplicated and the representative data are shown.
